# Comprehensive characterization of non-cellulosic recalcitrant cell wall carbohydrates in unhydrolyzed solids from AFEX-pretreated corn stover

**DOI:** 10.1186/s13068-017-0757-5

**Published:** 2017-03-29

**Authors:** Christa Gunawan, Saisi Xue, Sivakumar Pattathil, Leonardo da Costa Sousa, Bruce E. Dale, Venkatesh Balan

**Affiliations:** 1 Biomass Conversion Research Lab (BCRL), Chemical Engineering and Materials Science, Michigan State University, 3815 Technology Boulevard, Lansing, MI 48910 USA; 2 DOE Great Lakes Bioenergy Research Center (GLBRC), Michigan State University, East Lansing, MI USA; 3 Complex Carbohydrate Research Center, University of Georgia, Athens, GA 30602 USA; 4 Oak Ridge National Laboratory, Biosciences Division, BioEnergy Science Center (BESC), Oak Ridge, TN 37830 USA; 5 Present Address: Mascoma, LLC (Lallemand Inc.), 67 Etna Road, Lebanon, NH 03766 USA

**Keywords:** Recalcitrant cell-wall glycans, High-solids loading enzymatic hydrolysis, Unhydrolyzed solids, Glycome profiling, Monoclonal antibody, Non-cellulosic polysaccharides, Carbohydrates linkages

## Abstract

**Background:**

Inefficient carbohydrate conversion has been an unsolved problem for various lignocellulosic biomass pretreatment technologies, including AFEX, dilute acid, and ionic liquid pretreatments. Previous work has shown 22% of total carbohydrates are typically unconverted, remaining as soluble or insoluble oligomers after hydrolysis (72 h) with excess commercial enzyme loading (20 mg enzymes/g biomass). Nearly one third (7 out of 22%) of these total unconverted carbohydrates are present in unhydrolyzed solid (UHS) residues. The presence of these unconverted carbohydrates leads to a considerable sugar yield loss, which negatively impacts the overall economics of the biorefinery. Current commercial enzyme cocktails are not effective to digest specific cross-linkages in plant cell wall glycans, especially some of those present in hemicelluloses and pectins. Thus, obtaining information about the most recalcitrant non-cellulosic glycan cross-linkages becomes a key study to rationally improve commercial enzyme cocktails, by supplementing the required enzyme activities for hydrolyzing those unconverted glycans.

**Results:**

In this work, cell wall glycans that could not be enzymatically converted to monomeric sugars from AFEX-pretreated corn stover (CS) were characterized using compositional analysis and glycome profiling tools. The pretreated CS was hydrolyzed using commercial enzyme mixtures comprising cellulase and hemicellulase at 7% glucan loading (~20% solid loading). The carbohydrates present in UHS and liquid hydrolysate were evaluated over a time period of 168 h enzymatic hydrolysis. Cell wall glycan-specific monoclonal antibodies (mAbs) were used to characterize the type and abundance of non-cellulosic polysaccharides present in UHS over the course of enzymatic hydrolysis. 4-*O*-methyl-d-glucuronic acid-substituted xylan and pectic-arabinogalactan were found to be the most abundant epitopes recognized by mAbs in UHS and liquid hydrolysate, suggesting that the commercial enzyme cocktails used in this work are unable to effectively target those substituted polysaccharide residues.

**Conclusion:**

To our knowledge, this is the first report using glycome profiling as a tool to dynamically monitor recalcitrant cell wall carbohydrates during the course of enzymatic hydrolysis. Glycome profiling of UHS and liquid hydrolysates unveiled some of the glycans that are not cleaved and enriched after enzyme hydrolysis. The major polysaccharides include 4-*O*-methyl-d-glucuronic acid-substituted xylan and pectic-arabinogalactan, suggesting that enzymes with glucuronidase and arabinofuranosidase activities are required to maximize monomeric sugar yields. This methodology provides a rapid tool to assist in developing new enzyme cocktails, by supplementing the existing cocktails with the required enzyme activities for achieving complete deconstruction of pretreated biomass in the future.

**Electronic supplementary material:**

The online version of this article (doi:10.1186/s13068-017-0757-5) contains supplementary material, which is available to authorized users.

## Background

Declining crude oil reserves and environmental concerns associated with greenhouse gas emissions due to petroleum products has provided an impetus to transition from the current fossil fuel scenario to a more sustainable renewable energy system [1]. Inedible plant biomass, also known as lignocellulosic biomass, includes agricultural residues, forestry residues, herbaceous, and woody crops. These are the most abundant sources of potential feedstocks for producing renewable liquid transportation fuels [2]. Structural carbohydrates from the plant cell walls represent a vast untapped energy source. Attempting to economically convert these carbohydrates to biofuels, particularly via the biochemical route, will be a step forward in creating a more sustainable liquid fuel for transportation. Significant research efforts have been undertaken in the field, particularly over the past few decades.

Cellulose, hemicelluloses, and pectins in the plant cell wall are embedded in a complex matrix with lignin. Plant cell walls are highly recalcitrant to biomass-degrading enzymes, which are responsible to cleave glycosidic bonds and produce monomeric sugars for fermentation [3, 4]. Obtaining high yield of monomeric carbohydrates at minimal enzyme loading is challenging and it is one of the key bottlenecks for obtaining cost-effective biofuels [5, 6]. Due to the recalcitrant nature of the cell wall, pretreatment is required for improving the access of enzymes to their substrates and improve the efficiency of biomass deconstruction [7–9]. Ammonia Fiber Expansion (AFEX™)^1^ is a pretreatment process in which ammonia reacts with biomass at elevated temperatures and pressures. Ammonia can be used in liquid or gaseous forms and about 97% of ammonia can be recovered and reused in the process [10–12]. The AFEX process loosens the plant cell wall ultrastructure, cleaving lignin–carbohydrate complexes (LCCs), partly relocating lignin to the surface of the cell wall, leaving behind porous structures that help to improve enzyme accessibility to carbohydrates [4, 13]. Due to their physiochemical nature, AFEX is most effective on grasses, including CS, switchgrass, sugarcane bagasse, and miscanthus. As CS is the most abundantly available feedstock in the United States, AFEX could be a promising option for biofuel production in the US [14–16].

Unlike acidic pretreatments which require a wash stream, AFEX pretreatment is a dry-to-dry process that keeps the carbohydrate composition unaltered and preserves most of the sugars intact in a single solid biomass stream [3, 8, 13, 17, 18]. The presence of hemicellulose and pectin, however, requires more complex enzyme cocktails relative to acidic pretreatments, where hemicellulases, pectinases, and other accessory enzymes must be added to cellulases to maximize overall sugar yields. Non-cellulosic polysaccharides, which account for 25–35% of plant cell walls, have branched cross-linkages with varying levels of substitution [19–22]. Thus, a higher degree of synergy between a diverse set of enzyme activities is required to completely depolymerize such complex and highly branched carbohydrate structures into monomeric sugars [23, 24]. Though enzyme cocktail complexity is increased for deconstructing ammonia-pretreated biomass, the overall enzyme loading required does not change significantly in relation to dilute acid pretreatment [9].

A recent study by Uppugundla et al. showed that inefficient sugar conversion is a problem for various thermochemical pretreatment technologies, including AFEX, dilute acid, and ionic liquid pretreatments [9]. Using the advanced commercial cocktails containing Cellic Ctec2, Cellic Htec2, and Multifect Pectinase with optimized ratio, nearly 22% of total carbohydrates from AFEX-pretreated biomass were left behind as polymeric and oligomeric sugars after 7 days of hydrolysis, at high enzyme loading (20 mg protein/g glucan) and solids loading (6% glucan loading). Due to these unconverted sugars, the biofuel production potential is significantly reduced, which negatively impacts the overall economics of the biorefinery [25]. This is a universal problem faced by researchers both for hardwood and grass substrates [9]. Recalcitrant cell wall polysaccharides not only resist depolymerization when commercial enzymes are used, but also block the accessibility of cellulases to cellulose. Such effect further reduces overall sugar conversion and therefore, it is important to study and understand how commercial enzyme cocktails can be modified for improving hydrolysis of such recalcitrant polysaccharides.

One approach for studying this problem is to rationally design the enzyme cocktail by understanding the limiting factors that contribute to oligosaccharide and polysaccharide accumulation. For example, if some of the required biomass-degrading enzymes are not present or present at low levels in the commercial enzyme cocktail, some glycosidic linkages will tend to accumulate during the hydrolysis process. Thus, by carrying out detailed composition analysis and identifying structural features of enriched recalcitrant cell wall components, one can rationally determine the enzymes that are limiting the hydrolysis process. To facilitate such fundamental understanding of recalcitrant cell wall components, we require rapid tools that provide in-depth structural information about non-cellulosic glycans at the molecular level. One of the methods currently available is glycome profiling. Glycan profiling takes advantage of a worldwide collection of more than 200 plant cell wall glycan-directed mAbs to evaluate the glycan composition of plant cell walls. These mABs enable monitoring of carbohydrate epitopes found in most major non-cellulosic cell wall glycans [26]. Recent studies have employed glycome profiling to better understand cell wall modifications in plant biomass during genetic modifications, biomass pretreatments, and microbial fermentations [26–34]. This information is essential to better understand the glycan linkages contributing to biomass recalcitrance and develop strategies for overcoming this problem.

**Fig. 1:**
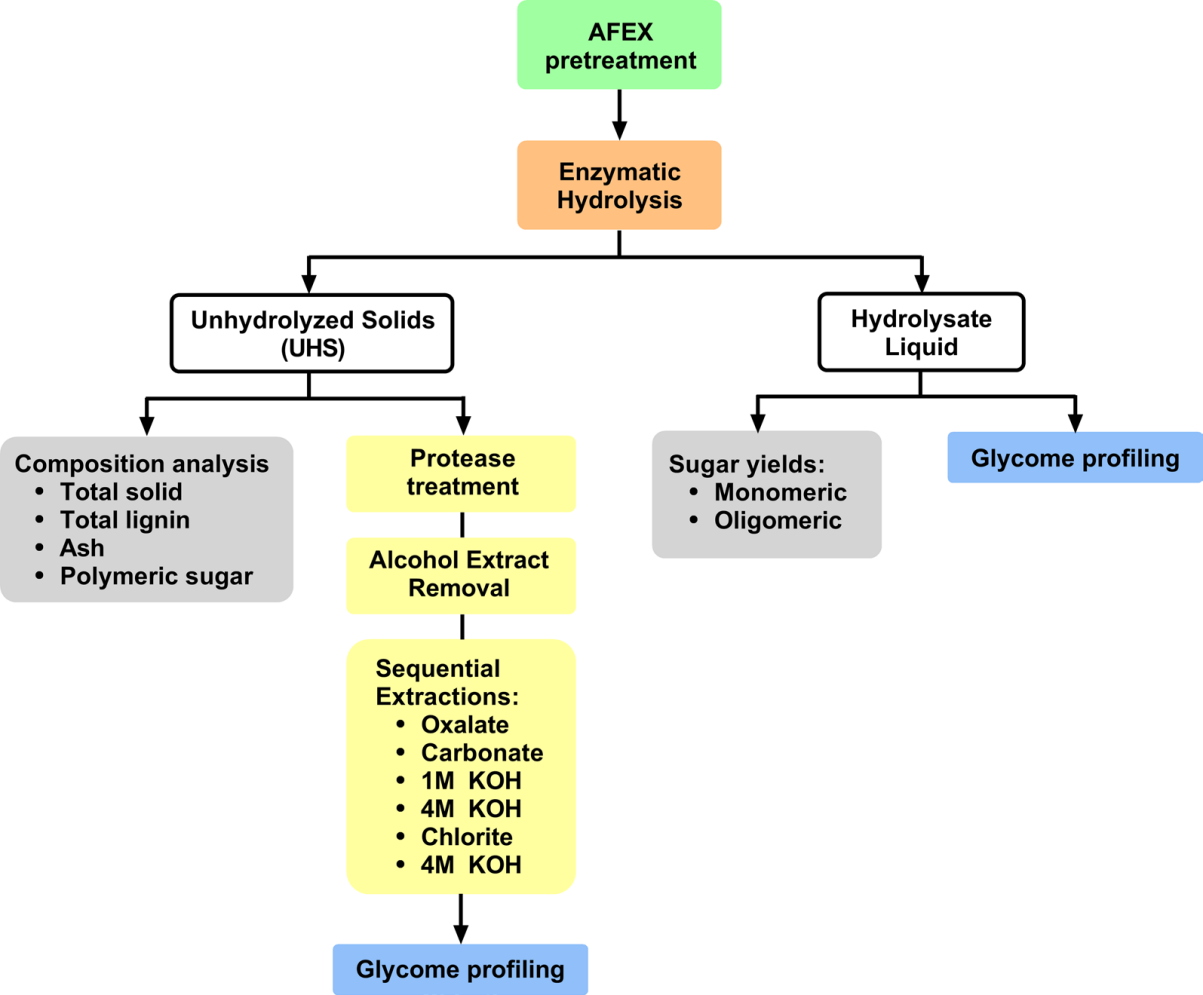
Process of characterizing the recalcitrant plant cell wall components in AFEX-CS

In this study, we used glycome profiling to identify the cell wall components that remain intact after prolonged enzymatic hydrolysis by commercial enzymes, including specific glycan residues from AFEX-pretreated corn stover (AFEX-CS) (Fig. 1). The glycan epitopes in both unhydrolyzed solids (UHS) and hydrolysates after high-solids-loading enzymatic hydrolysis were analyzed in order to determine which groups of polysaccharides are most abundant and resistant to commercial enzyme cocktails. To our knowledge, this is the first study using glycome profiling to understand unhydrolyzed cell wall constituents present in UHS and liquid hydrolysate after intensive enzymatic hydrolysis. The structural information obtained from this study provides insights about important enzyme activities that are needed to make better commercial enzyme cocktails, compared to the cocktail used in this study. Improved cocktails will help increase sugar conversion during enzyme hydrolysis, increase biofuel yield, and reduce biofuel cost in a biorefinery.

## Results and discussion

### Time profile of unhydrolyzed solids (UHS) composition

**Fig. 2:**
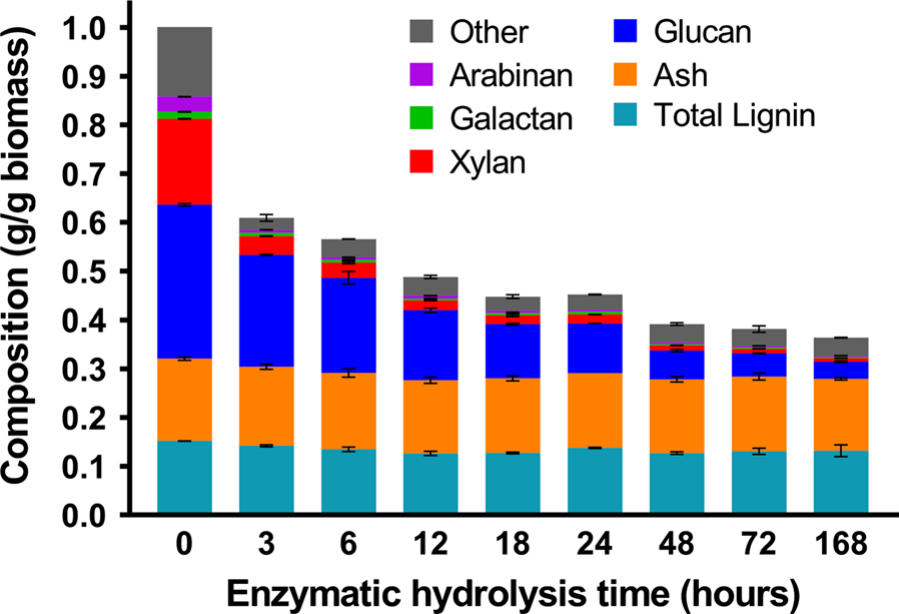
Composition of insoluble solids throughout enzymatic hydrolysis. Total height is normalized to the original amount of CS prior to hydrolysis. Composition includes all the insoluble structural carbohydrate, combined acid soluble and insoluble lignin, and ash

**Fig. 3:**
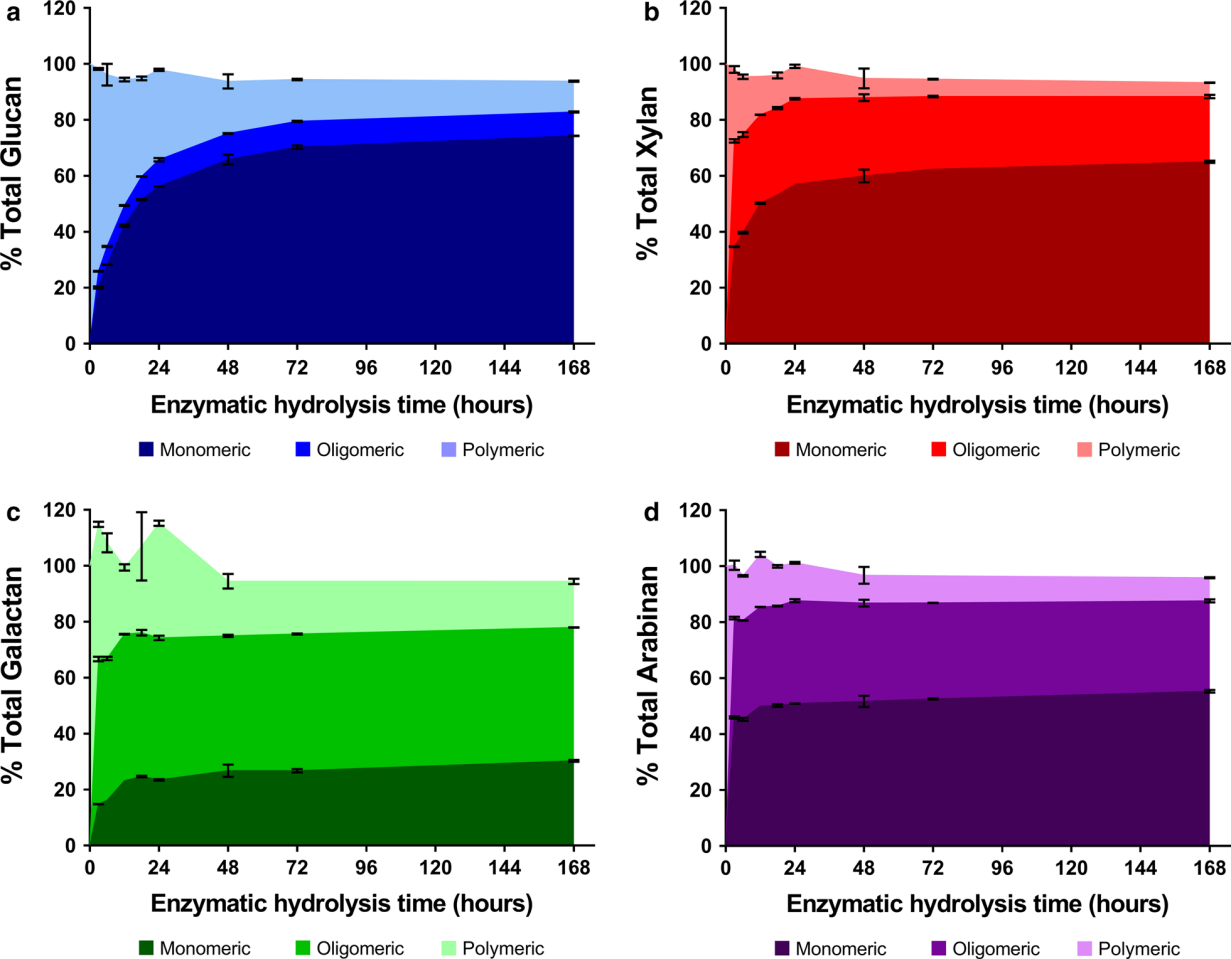
Mass balance for glucan (**a**), xylan (**b**), galactan (**c**), arabinan (**d**) throughout the course of hydrolysis. Monomeric and oligomeric sugars are measured in the liquid portion, while polymeric sugar is measured in the unhydrolyzed insoluble material. Total *shaded area* represents mass balance closure. The *Y*-*axis* has been scaled to 100% as the maximum at the beginning of hydrolysis

To understand how cellulose and hemicellulose-derived sugars are released from AFEX-CS during the course of enzymatic hydrolysis, the composition of UHS was periodically analyzed during 168 h hydrolysis. The details about mass balance for AFEX pretreatment and enzyme hydrolysis can be found in previously published work [9]. The amount of insoluble solids continuously decreased throughout the course of hydrolysis as shown in Fig. 2. The hemicellulose, which includes xylan, arabinan, and galactan, was rapidly hydrolyzed, decreasing from 0.22 g/g CS at the start of hydrolysis to 0.05 g/g CS within the first 3 h. This dramatic reduction shows that the majority of the digested hemicellulose was converted into soluble sugar (i.e., oligomers and monomers). The hemicellulose polysaccharides further decreased to 0.03 g/g CS after 24 h. During the remaining 6 days period of hydrolysis, little amounts of hemicellulose polysaccharides were further solubilized. In contrast, the reduction in cellulose content was more gradual throughout the entire course of hydrolysis, whereas the amount of insoluble lignin and ash (which includes soil that is brought in with the biomass) remained practically constant. These results were confirmed by the mass balance summarized in Fig. 3, where monomeric and oligomeric sugars present in the liquid phase (the hydrolysate) and the insoluble polysaccharides present in the solid phase (UHS) throughout the course of hydrolysis are shown. All results are normalized as a percentage of the amount present in the UHS and a mass balance closure over 95% was obtained for all sugars analyzed in this study. The combined amount of soluble (oligomeric and monomeric) hemicellulose, including xylose, arabinose, and galactose, reached 70–80% of the total sugars within the first 3 h. The amount of monomeric hemicellulose-derived sugars continued to increase slightly throughout the rest of the hydrolysis, although the total soluble sugars remained constant between 24 and 168 h. In contrast, mostly glucose monomers were produced within the first 24, which continued to increase slowly for the rest of the 6-day period. Glucose oligomers also increased slowly over the course of hydrolysis, while hemicellulose-derived oligomers decline slowly. These results are consistent with other studies showing that most biomass is solubilized within the first 24 h of enzyme hydrolysis under high enzyme loading hydrolysis. Also, it confirms the differences between cellulose and hemicellulose degradation patterns by enzymes [9, 35].

Among the soluble sugars, hemicelluloses are generally more difficult to convert to monomeric sugars and therefore, we observed considerable levels of xylan, galactan, and arabinan containing oligomers during enzymatic hydrolysis. Nearly 25% of the total xylan was present as oligomers after 72 h, while only 9% of the glucan is present as oligomers at this time point. Likewise, a large proportion of arabinan and galactan sugars remained as oligomers, as shown in Fig. 3c and d. Most of the recalcitrant carbohydrates in hemicellulose (xylan, arabinan, and galactan) were solubilized as oligomers. It appears that the commercial enzymes used here can efficiently solubilize some of the hemicelluloses, but unable to cleave all the hemicellulose linkages to generate monomeric sugars. Cellulose is a relatively simpler structure consisting of glucan chains connected with inter- and intra- molecular hydrogen bonding. However, hemicellulose is highly branched with multiple sugars and cross-linked with other organic moieties (e.g., acetyl, feruloyl, galacturonic, glucoronyl), some of which form complexes with lignin [36]. Multiple accessory enzymes are required to fully break down these complex hemicellulose linkages [37]. Based on our results, it is unlikely that all those enzymes are present at sufficient quantities and/or activities in the commercial enzyme cocktails used in this study. To determine the most abundant linkages present in UHS after enzymatic hydrolysis, we have further performed glycome profiling on UHS produced during enzymatic hydrolysis of AFEX-CS. As control experiments, we have also performed glycome profiling of untreated and AFEX-CS.

### Glycome profiling of untreated and AFEX-pretreated biomass

**Fig. 4:**
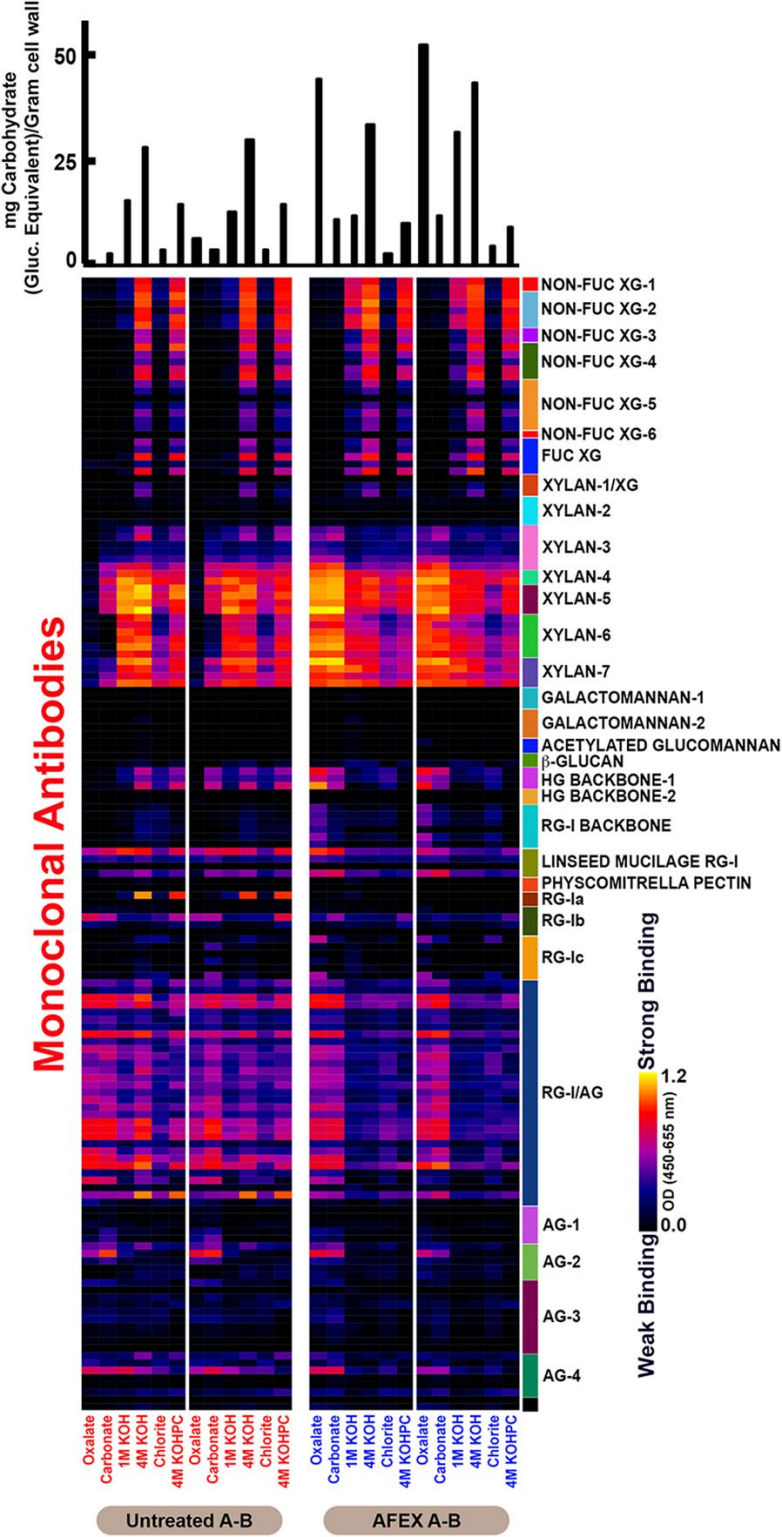
Glycome profiling of the cell wall extracts of untreated CS versus AFEX-CS. Here, *A*-*B* represents replicates of untreated or pretreated biomass prior to hydrolysis. *Labels at the bottom of each panel* indicate the reagents used for the sequential extractions of the cell wall. The amounts of sugars extracted are shown in the *bar graphs* above the panel. All the antibody groups used for the ELISA screening are shown on the right side of the heat map

**Fig. 5:**
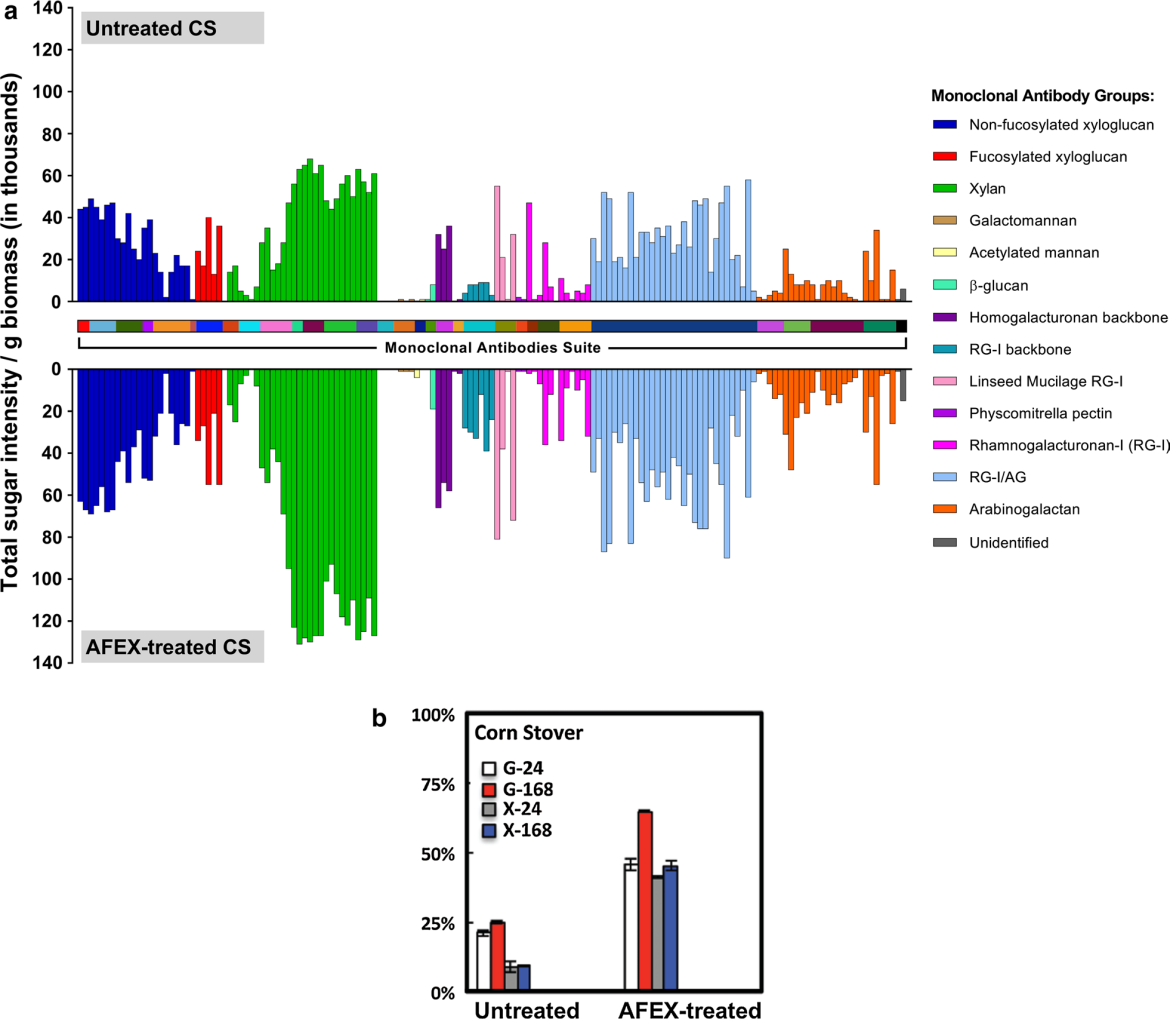
AFEX increases the extractability (**a**) and digestibility (**b**) of CS. Here, **a** extractability is measured by total sugar intensity from all extracts from sequential extractions. On the top is the untreated CS and in the bottom is the AFEX-CS. The *Y*-*axis* shows the ratio of total sugar intensity versus g of biomass in thousands (*1000). All antibody groups used for ELISA screening are shown on the right side of the heat map. **b** Digestibility of untreated and AFEX-CS as total glucan-to-glucose yields after 24 and 168 h are shown in white and red bars, respectively. Total xylan-to-xylose yields after 24 and 168 h are shown in gray and blue bars, respectively. *Error bars* depict standard deviations of data from the mean values reported for assays conducted in triplicate (Adapted from S. Pattathil et al. [31]; Fig. 7)

Prior to glycome profiling, cell wall materials were prepared from biomass residues and subjected to six sequential extractions with reagents of increasing severity, notably ammonium oxalate (50 mM), sodium carbonate (50 mM), KOH (1 and 4 M), and acidic chlorite. These reagents selectively solubilize cell wall matrix polysaccharides on the basis of the relative tightness with which they are integrated into the plant cell walls. The extracts were then subjected to ELISAs against a comprehensive suite of 155 cell wall glycan-directed mAbs, providing responses that were further represented as heat maps. Hierarchical clustering of binding data for these mAbs against 54 structurally known plant polysaccharides allowed classification of these mAbs into the categories used in this work [38]. In previous glycome profiling studies [31], it has been demonstrated that AFEX pretreatment significantly reduces cell wall recalcitrance by inducing structural modifications to the polysaccharide network. In Fig. 4, AFEX pretreatment induced enhancement in the extractability of non-cellulosic cell wall glycans including xylans and pectins, as indicated by the increased binding of specific groups of mAbs, notably xylan-3 through xylan-7 and pectic backbone (HG backbone-I and RG-I backbone) groups of mAbs, to oxalate and carbonate extracts from AFEX-CS (oxalate and carbonate extractions are performed in milder conditions and therefore, extracted glycans are more loosely bound to lignin compared to KOH and chlorite extracts). Normalized data of the sugar intensity (gram per gram of biomass in thousands) (Fig. 5a) allow a closer examination of the different epitopes’ extractability from AFEX-CS relative to untreated CS. From Fig. 5a, it is clear that the overall intensity of extracted carbohydrates in the untreated samples is much lower than those from AFEX-CS for most epitopes, especially for the xylans. From previous reports we know that AFEX pretreatment partially solubilizes hemicellulose, loosens the cell wall, and cleaves lignin–carbohydrate complexes [4, 13]. The cell wall modifications that take place during pretreatment increases enzyme access to cellulose and hemicelluloses chains for subsequent depolymerization. Thus, the increased extractability of major non-cellulosic glycans is thought to be associated with loosening of the cell wall structure, resulting in better enzyme access and digestibility after AFEX pretreatment (Fig. 5b). This observation may also help explain the rapid hemicellulose solubilization within the first 3 h of enzymatic hydrolysis, as observed in Fig. 3 b–d. The greatly improved digestibility of AFEX-CS during animal feed trials also supports the hypothesis that hemicellulose is quickly digested by ruminant microorganisms, allowing cellulose to be increasingly exposed for subsequent degradation and bioconversion. As a result, AFEX-CS releases more energy and nutrients to ruminant animals compared to untreated CS [39, 40].

### Glycome profiling and structural insights of UHS

**Fig. 6:**
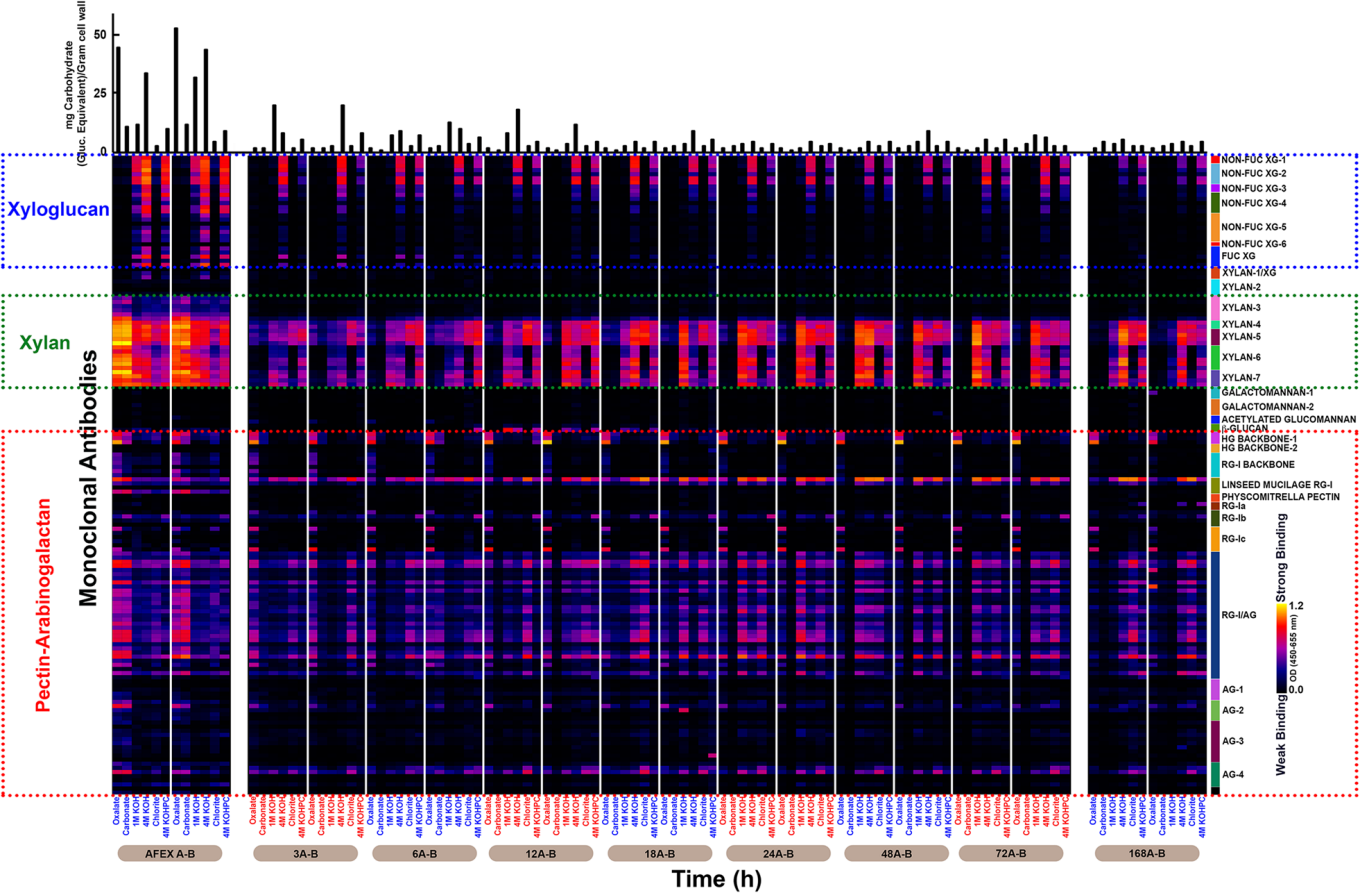
Glycome profiling of the cell wall extracts of AFEX-CS over the course of hydrolysis. Here, *A*-*B* represents replicates of extracts. AFEX *A*-*B* showed the composition of biomass at the beginning of hydrolysis. The other *panels* show the time points at which UHS were sampled (*3A*-*B* indicates 3 h time point). *Labels at the bottom of each panel* indicate the reagents used for the sequential extractions of the cell wall. The amounts of sugars extracted are shown in the *bar graphs* above the *panel*. All the antibody groups used for the ELISA screening are shown on the *right side* of the heat map

In order to elucidate the overall composition and extractability of non-cellulosic cell wall glycans that remained insoluble in UHS after enzymatic hydrolysis of AFEX-CS, glycome profiling was applied to UHS as a function of hydrolysis time (Fig. 6). Overall, fewer carbohydrates were recovered in extracts from UHS subjected to prolonged enzymatic hydrolysis (see bar graphs on the top of Fig. 6 for sugar extracted per gram of cell wall in each step). Compared with AFEX-CS, UHS produced in the first 3 h of enzymatic hydrolysis showed significantly lower carbohydrate recovery among various cell wall extracts, especially hemicellulose and pectins (xylans and pectic arabinogalactans, respectively). This observation is consistent with the results shown in Fig. 3, where most of the hemicelluloses in the plant cell wall were solubilized within the first 3 h of enzymatic hydrolysis. After 3 h enzyme hydrolysis, a significant amount of xyloglucan and xylan epitopes were converted including the epitopes recognized by non-fucosylated xyloglucan-3 through non-fucosylated xyloglucans-6, fucosylated xyloglucans, and xylan-1 though xylan-3 groups of mAbs. These epitopes completely disappeared after 12 h hydrolysis. Following the same pattern, epitopes recognized by mAbs against RG-I backbone were also converted gradually with time, completely disappearing from the ELISA heat map after 24 h of hydrolysis. In all the UHS analyzed in this study, most of the xylan epitopes were not detectable in oxalate and carbonate extracts, revealing that the easily extractable xylans from AFEX-CS, which are not strongly associated with lignin and/or other insoluble cell wall polymers, were mostly digested within the first 3 h of hydrolysis. This observation supports the hypothesis that the more loosened cell wall components that can be extracted under milder conditions are more accessible to enzymes and therefore, they can be more easily digested. However, xylan epitopes recognized by xylan-4 through xylan-7 groups of mAbs were still present in the oxalate and carbonate extracts after 3 h of hydrolysis, and were further enriched for the harsher extraction conditions (1 M KOH, 4 M KOH, and chlorite treatment followed by 4 M KOH) and after 168 h of hydrolysis. We would like to emphasize that this is a key observation, as it indicates that some highly substituted xylan components in AFEX-CS cannot be completely deconstructed with current state-of-the-art commercial enzyme cocktails. When associated with lignin and/or other insoluble cell wall components, these substituted xylans tend to be even more resistant to hemicellulase enzymes. Lignin, which is enriched during enzymatic hydrolysis, acts as a barrier for enzymes to access these carbohydrate linkages, which only become accessible to the mAbs after a harsh base treatment. Apart from xylan epitopes, those comprising pectic-arabinogalactan, arabinogalactans, and non-fucosylated xyloglucans, also remained present in oxalate and alkaline extracts after 168 h enzymatic hydrolysis (Fig. 6).

The results from Fig. 6 show that great part of the undigested epitopes present in UHS is only revealed after alkaline or chlorite treatment. Those carbohydrates are typically secondary cell wall components, which are still coupled with lignin. Some of those specific carbohydrates may be totally blocked by the presence of lignin, which does not allow enzymes to access their substrates [41], resulting in a significant epitope accumulation for various groups of polysaccharides (Fig. 6). Some of the epitope linkages have been identified in previous work. For example, xylan-5 mAb recognizes one of the most recalcitrant non-cellulosic glycans present in UHS and hydrolysate [42, 43]. The epitope for this mAb contains a 4-*O*-methyl glucuronic acid side residue on an otherwise linear xylan backbone (Table 1) [44]. It appears that this particular side chain is poorly cleaved during the hydrolysis process, indicating low α-glucuronidase activity. Likewise, RG-1/AG epitopes correspond to pectic-arabinogalactan linkages. This is consistent with Fig. 3, in which ~50% of the galactan remains in the oligomeric form through enzymatic hydrolysis. Linseed mucilage RG-1 mAb, which is associated with rhamnogalacturonan-I, had the strongest binding in UHS from AFEX-CS. Rhamnogalacturonan-I often has arabinan and galactan side chains, requiring multiple enzyme activities to be fully decomposed to monomers [45–47]. Identifying appropriate accessory enzymes that can cleave these side chain residues is required for achieving complete deconstruction of these complex carbohydrates. We hypothesize that the commercial enzyme cocktail used in this work requires those supplemental enzyme activities, which will synergize and significantly increase hemicellulose conversion during enzymatic hydrolysis of AFEX-CS. Increasing the levels of these missing enzymes may also increase the rate and extent of cellulose hydrolysis by unmasking cellulose chains more readily and making them more accessible to cellulase enzymes.

**Table 1:**
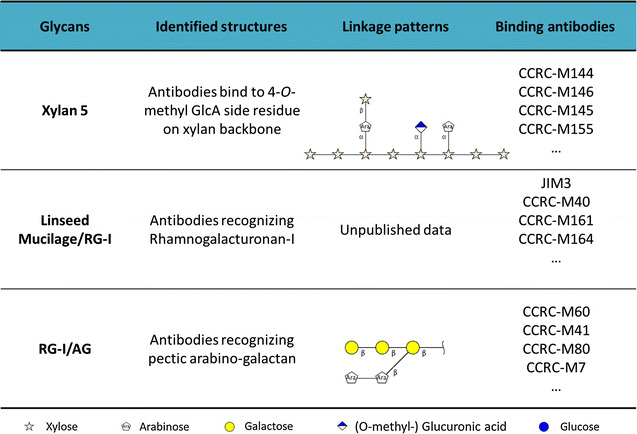
Antibodies-binding epitopes of the most recalcitrant glycans and their cross-linkage patterns. Linkages were depicted with GlycoWorkBench developed in CCRC-UGA

Although the heat map from Fig. 6 indicates the glycans that remain present in UHS during enzymatic hydrolysis, the intensity shown is for a constant amount of extracted sugars rather than representing the relative amount of sugar present as a function of time. When normalized to the initial amount of epitope present in AFEX-CS (Table 2), the total amount of each epitope present in UHS as a function of time correlates to the trends of carbohydrate solubilization seen in Fig. 2. Almost all the non-cellulosic polysaccharide components rapidly decreased within the first 3 h, further decreasing to levels below 4% of their initial value after 24 h enzymatic hydrolysis. The fact that some non-cellulosic glycan linkages remain insoluble throughout hydrolysis could suggest that they may be completely resistant to enzyme digestion by the commercial enzymes used in this study, completely surrounded by lignin (blocking enzyme access) or enzymes could be inhibited. Looking closer to the hydrolysate we could find some evidence that lignin blockage may not explain everything about the recalcitrance of the UHS carbohydrates. In Table 2 and Additional file 1: Figure S1 (see ESI), it is clear that the liquid hydrolysate contains xylan-5, Linseed Mucilage/RG-I, and RG-I/AG mAb-groups detected epitopes, suggesting that the commercial enzymes were not able to hydrolyze those linkages, even when they are accessible as soluble oligosaccharides, without the presence of enriched insoluble lignin. The reduction of xylan-5, Linseed Mucilage/RG-I, and RG-I/AG epitopes in the UHS over the course of enzymatic hydrolysis could potentially be attributed to solubilization of carbohydrate fragments containing those epitopes. The presence of these soluble epitopes in the liquid hydrolysate is likely due to lack of enzyme activity, either by the absence or presence of low levels of specific enzymes, or enzyme inhibition. In all these cases, it is important to increase the ratio of enzymes that could break those epitopes, so that complete conversion of those soluble oligosaccharides to fermentable sugars can be achieved. It is also important to mention that the current glycome profiling method is only effective for detecting oligosaccharides of DP larger than 20 and our previous work has shown that most oligosaccharides have DP lower than 20 [22]. Therefore, it is likely that there are other undigested epitopes that could not be detected by this method. For overcoming this limitation and to have a better representation of the undigested epitopes in solution, the current glycome profiling technique must be modified.

**Table 2:**
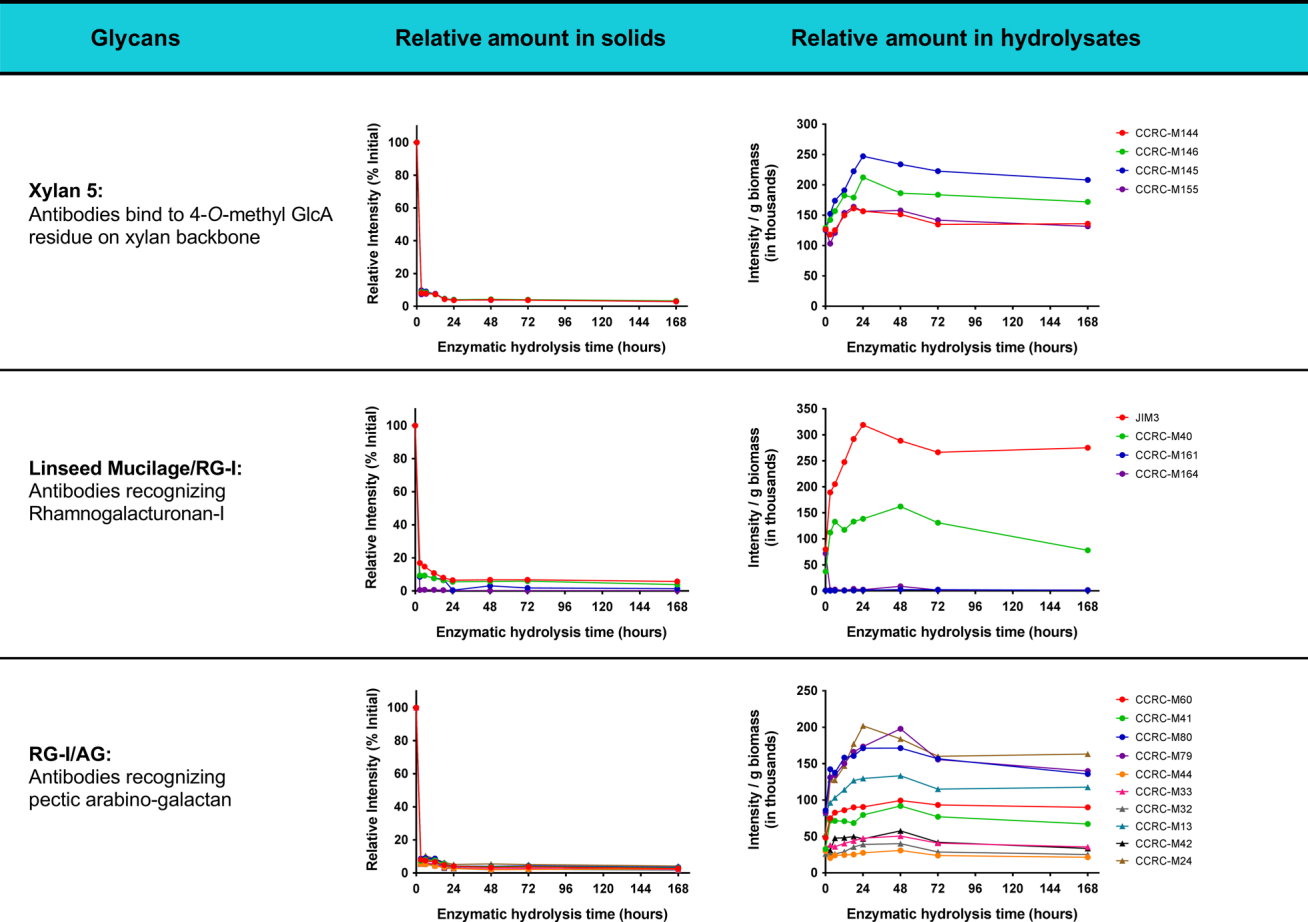
Relative amount of sugar normalized to the amount of AFEX-CS present in each stage of hydrolysis present at each time point

Overall, the information provided by this work shows that specific glycans (e.g., xylans decorated with 4-*O*-methyl glucuronic acid residues, pectic arabinogalacta, and rhamnogalacturonan-I) are not completely digested by the commercial enzyme cocktail used in this work, even when those epitopes are completely soluble in the liquid hydrolysate and potentially free from lignin blockage. In contrast, a larger range of glycan epitopes can be detected in the UHS when they are associated with lignin, suggesting that those glycans may not be accessed by enzymes due to lignin blockage. Though these insoluble non-cellulosic carbohydrates represent less than 7% of the total carbohydrates after excessive enzyme treatment with optimized cocktails, the soluble counterpart in hydrolysate represents as much as 15% of the total carbohydrates in pretreated biomass, which is a much more significant fraction of substrate that is not converted to monomeric sugars and biofuel, ultimately. In future work, our goal is to better understand the factors that contribute to epitope accumulation in the UHS and liquid hydrolysate. Imaging techniques, such as TEM and florescent microscopy, can be applied to depict the spatial orientation of cell wall components in the UHS and understand phenomena such as lignin blockage. NMR and mass spectrometry can also be used to determine the composition, structure, and linkage patterns of purified recalcitrant carbohydrates (mostly oligosaccharides). All these studies will complement this work and help to comprehensively understand cell wall recalcitrance. The information provided by this (and future) work will help us to rationally redesign the enzyme cocktail, by adding a selection of key enzymes for improving monomeric sugar yields, with minimal enzyme usage.

## Conclusions

The chemical nature of some of the recalcitrant carbohydrate linkages present in CS was studied by analyzing UHS and hydrolysates resulting from enzymatic hydrolysis of AFEX-CS. Samples taken at multiple time points over a period of 168 h were analyzed to understand changes in the different cell wall components as a function of time. The polysaccharides that were easier to extract after AFEX treatment were rapidly deconstructed by the enzymes, while some of the carbohydrates that required harsher alkaline extractions could not be hydrolyzed by the enzymes and accumulated in the UHS. While a wide range of polysaccharides remained in the UHS, the amount remaining in the insoluble form was relatively small (<5%) after 168 h. However, soluble polysaccharides, particularly those recognized by xylan-5 and RG-I/AG groups of mAbs, remained abundant in hydrolysate and UHS throughout the course of hydrolysis, indicating a lack of appropriate enzyme activities or severe enzyme inhibition. These results show that complete sugar conversion is not possible when using commercial enzyme cocktails (Cellic Ctec2, Cellic Htec2, and Multifect Pectinase) as used in this work, at high-solid loading enzymatic hydrolysis conditions.

Future work is needed to find enzymes that hydrolyze these recalcitrant non-cellulosic polysaccharide linkages. For example, accessory enzymes such as pectinase, α-glucuronidase, and enzyme activities targeting arabinan and galactan should be identified and added to the enzyme cocktail, so that branched linkages that block enzyme accessibility to backbone polysaccharide chains can be hydrolyzed and potentially be decoupled from the complex structure of hemicellulose. If successful, this approach could not only increase monomeric xylose yields, but may also synergistically improve cellulose hydrolysis, thus increasing glucose yields and a possible reduction in enzyme loading to lower biofuel production cost. Likewise, this approach could be adapted with other pretreatment technologies and biomass to optimize hydrolysis conditions for maximum sugar output. Moreover, since routine glycome profiling only detects large polysaccharides, more advanced techniques such as biotinylation [48], fluorescent-labeled antibodies studies using fluorescent microscopy and flow cytometry will be able to detect short-chain oligosaccharides that are abundant in the hydrolysate. Such studies are underway at the Great Lakes Bioenergy Center (GLBRC).

## Methods

### Corn stover and AFEX pretreatment

The corn (Pioneer 36H56) was planted on May 20, 2010 in field 436 of Arlington Agricultural Research Station, Wisconsin. The field was fertilized with 340 lbs/acre urea 3 days prior to planting. In October 22, 2010, the CS was harvested and milled to a particle size of 5 mm. AFEX pretreatment was performed on the CS at 100 °C for 30 min with 0.6 g ammonia and 1 g water per g biomass loading in a bench-top stainless steel batch reactor (Parr Instruments Company) [10, 11, 13]. It took 30 min for the reactor to reach 100 °C and this condition was maintained for 30 min. Then the ammonia was rapidly released, which immediately brought the biomass to room temperature. After the treatment, the biomass was transferred to aluminum tray and kept in hood overnight to remove residual ammonia, leaving behind dry material. The AFEX-CS contained 31.4% glucan, 18.7% xylan, 1.4% galactan, 3.3% arabinan, 14.3% lignin (1.23% acid soluble lignin, absorption wavelength 320 nm, absorptivity coefficient 30 L/g cm), and 13.4% ash.

### Enzymatic hydrolysis

Enzymatic hydrolysis was performed in duplicate using baffled Erlenmeyer flasks. The AFEX-CS was loaded at 20% dry solids in a fed-batch manner. Half of the biomass was loaded at *t* = 0 h, and the remaining biomass was loaded at *t* = 45 min. Commercial enzymes Cellic^®^ CTec2 (Novozymes), Cellic^®^ HTec2 (Novozymes), and Multifect Pectinase (Genencor) were loaded at 10, 5, and 5 mg protein/g glucan at *t* = 0 h, respectively. Flasks were incubated in a shaking incubator set at 250 rpm and 50 °C. During the sampling process, the flasks were taken out of the incubator and immediately set on ice for approximately 30 min to arrest the hydrolysis reaction at each time point (3, 6, 12, 18, 24, 48, 72, and 168 h). Separate pairs of flasks were used for each time point. The pH was adjusted to 5.0 using 12 M hydrochloric acid at the start of the hydrolysis process.

### Post-hydrolysis solids recovery

The contents of the flasks were transferred into centrifuge bottles and centrifuged at 10,000×*g* at 4 °C for 30 min. The supernatant was decanted, the volume measured, and filtered through 0.22 μm PES membrane and stored at 4 °C for future sugar analysis. The solid was re-suspended in a known amount of water (approximately 8:1 water-to-solid ratio) and centrifuged. The supernatant was decanted to a separate tube and a ~1 mL sample was taken for sugar analysis. This process was repeated two more times to remove any residual soluble material present in the solids. The moisture content of the wet solids was measured in triplicate by drying samples at 110 °C overnight in aluminum tray.

A portion of the washed solid was treated with protease from *Streptomyces griseus* (Sigma Aldrich P5147) at 5% (w/v) solid loading according to the procedure by Berlin et al. [41]. This helped to remove protein and residual enzymes bound to the cell walls prior to glycome profiling. The remaining solid was freeze-dried and stored in a refrigerator for further analysis.

### Liquid and solid composition analysis

The hydrolysate supernatants were diluted and analyzed for monomeric and oligomeric sugar contents. Monomeric sugars were analyzed using an HPLC equipped with a Bio-Rad (Hercules, CA) Aminex HPX-87P column and de-ashing guard column. Column temperature was held at 80 °C, and water was used as the mobile phase flowing at 0.6 mL/min. Oligomeric sugars were determined via dilute acid hydrolysis at 121 °C according to the method of Sluiter et al. [49]. Hydrolysis samples were neutralized and analyzed using the HPLC method given above for total sugars estimation following acid hydrolysis. The oligomeric sugars were calculated as total sugars after subtracting the monomeric sugars present in hydrolysate.

Freeze-dried solids were homogenized using mortar and pestle. Composition analysis was performed on the solids using the standard National Renewable Energy Laboratory (NREL) method for determination of structural carbohydrates and lignin according to Sluiter et al. [50].

### Mass balance

Mass balances were performed on the major structural components of CS cell walls at different time points throughout the course of the enzymatic hydrolysis process. All mass balances were based on 1 g of AFEX-CS. Total UHS were collected and measured as described above, while the liquid supernatant was analyzed for monomeric and oligomeric sugars. Because the hydrolysis was performed at a high solid loading, the volume of liquid was not constant throughout hydrolysis and was measured or calculated at the end of hydrolysis. The volume of the hydrolysate was calculated using the following equation:

$$ V_{\text{H}} = V_{{\text{S }}} + \frac{{V_{\text{w}} \times C_{\text{w}} }}{{C_{\text{H}} - C_{\text{w}} }}, $$

where *V*_H_ is the total volume of hydrolysate to be calculated; *V*_S_ is the measured volume of the supernatant of the hydrolysate; *V*_w_ is the measured amount of water added to the first wash step (as described in the post-hydrolysis recovery section above); *C*_w_ is the glucose concentration of the washed supernatant; and *C*_H_ is the glucose concentration of the hydrolysate. Here glucose was used to calculate the volume as it was the most abundant sugar.

### Glycome profiling

Glycome profiling of untreated, AFEX™-pretreated and all unhydrolyzed biomass residues (involving preparation of sequential cell wall extracts and their mAb screenings) was carried out using the SOP previously described [29, 33]. To conduct glycome profiling, Alcohol Insoluble Residue (AIR) cell wall materials were prepared from biomass residues and were subjected to sequential extractions with increasingly harsh reagents such as ammonium oxalate (50 mM), sodium carbonate (50 mM), KOH (1 and 4 M), and acidic chlorite as explained previously [33]. The extracts were then subjected to ELISAs against a comprehensive suite of cell wall glycan-directed mAbs [33] and the mAb binding responses were represented as heat maps. The amounts of different cell wall materials recovered during each extraction are depicted as bar graphs above the respective heat map panels. Plant cell wall glycan-directed monoclonal antibodies (mAbs) were received from laboratory stocks (CCRC, JIM and MAC series) maintained by the Complex Carbohydrate Research Center (available through CarboSource Services; http://www.carbosource.net) or were obtained from Bio-Supplies (Australia) (BG1, LAMP). Information on mAbs used in this study can be found in Table 2, including the link to Wall MabDB (http://www.wallmabdb.net) that provides detailed information for each antibody.

### Additional file

**Additional file 1: Figure S1.** Glycome profiling of the hydrolysates of AFEX-CS over the course of hydrolysis. A-B represents replicates of hydrolysate samples. Antibody groups used for the ELISA screening are shown on the right side of the heat map.

## Abbreviations

AFEX: Ammonia Fiber Expansion
CS: corn stover
UHS: unhydrolyzed solids
mAbs: monoclonal antibodies

## Footnotes

1 TM-AFEX is a trademark of MBI International, Lansing, Michigan.
