# Supplementary figures and images for: Comprehensive characterization of non-cellulosic recalcitrant cell wall carbohydrates in unhydrolyzed solids from AFEX-pretreated corn stover
Source: Biotechnol Biofuels. 2017 Mar 29;10:82. doi: 10.1186/s13068-017-0757-5 (PMC5372267; doi:10.1186/s13068-017-0757-5)

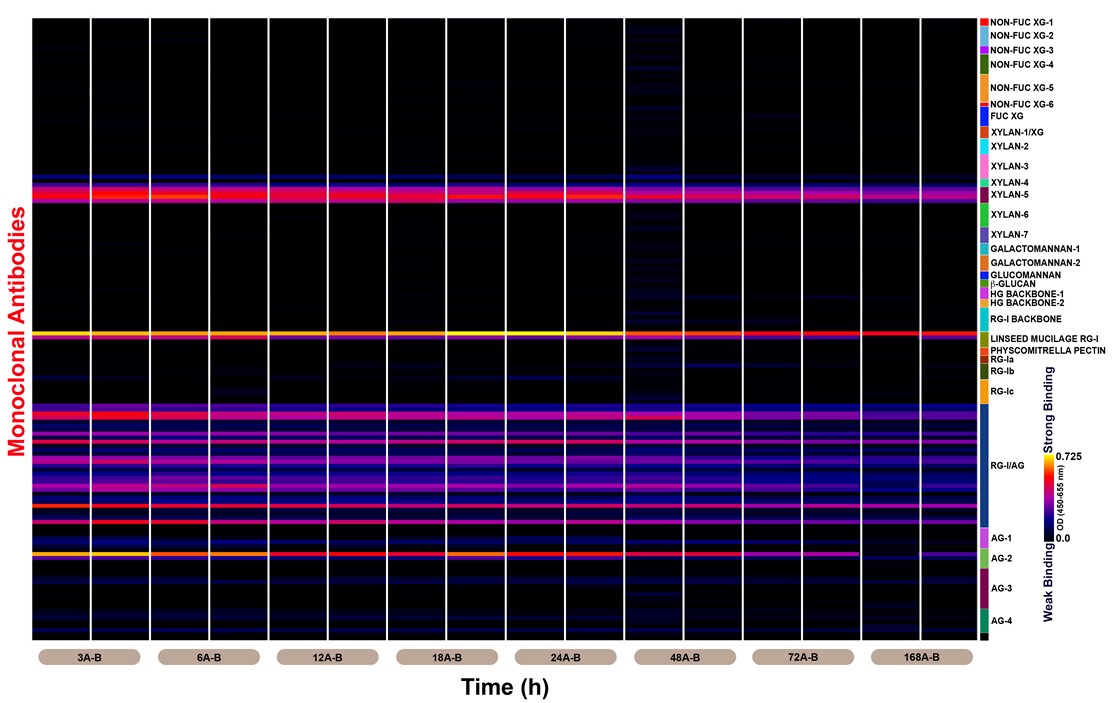

Supplement: Supplementary file 1 — Additional file 1: Figure S1. Glycome profiling of the hydrolysates of AFEX-CS over the course of hydrolysis. A-B represents replicates of hydrolysate samples. Antibody groups used for the ELISA screening are shown on the right side of the heat map. [file 13068_2017_757_MOESM1_ESM.jpg]
